# Is there a link between non-steroidal anti-inflammatory drugs and cardiovascular risk in patients with inflammatory arthritis?

**DOI:** 10.1016/j.athplu.2025.12.002

**Published:** 2026-03-11

**Authors:** Gerhard Zingler, Christoph Baerwald

**Affiliations:** a MSD Sharp & Dohme GmbH, Medical Department, Munich, Germany, Sonnenblumenweg 76, 18119, Rostock, Germany; b Zentrum für Seltene Erkrankungen, University Hospital Leipzig, Philipp-Rosenthal-Str. 55, 04103, Leipzig, Germany

**Keywords:** Cardiovascular risk, NSAIDs, Systemic inflammation, Osteoarthritis, Rheumatoid arthritis, Major cardiovascular events (MACE)

## Abstract

Non-steroidal anti-inflammatory drugs (NSAIDs) require particular attention, as these pharmaceutical compounds have been associated with a range of adverse effects, such as renal and gastrointestinal toxicity. In addition, data suggest an increased cardiovascular (CV) risk in the general population, which indicates a need for caution regarding its application in patients with elevated CV risk. Patients with inflammatory arthritis demonstrate an inherent predisposition to an elevated risk of CV morbidity and mortality. In this publication, we examine the available data indicating that the utilization of NSAIDs among patients diagnosed with inflammatory joint disease is not associated with an elevated risk of developing CV disease. The analgesic and anti-inflammatory properties of NSAIDs are discussed as a potential underlying mechanism contributing to a cardioprotective effect. In this context, patients diagnosed with rheumatoid arthritis (RA) or ankylosing spondylitis (AS), who also exhibit systemic inflammation, have been observed to benefit in respect to CV events in response to utilization of NSAIDs.

## Highlights

• Patients with inflammatory arthritis (IA) exhibit a predisposition to an elevated risk of CV morbidity and mortality.
• The utilization of NSAIDs or coxibs in patients diagnosed with IA requires a re-evaluation concerning CV risk.
• Available data indicate that NSAIDs and coxibs promote a protective effect for CV events in IA patients.
• The anti-inflammatory effect of NSAIDs is most probably the relevant factor in reducing CV events in IA and OA patients.

## 1 Introduction

In 1998, the approval of two tumour necrosis factor-α inhibitors (TNFi), infliximab and etanercept, marked the beginning of a successful development phase in treating chronic systemic inflammatory rheumatic diseases. This ongoing phase has resulted in significant advances in research and treatment [1]. Data from the Oslo RA registry indicate that coordinated treatment strategies, which involve intensified therapy after diagnosis and timely escalation of medication using methotrexate and TNF inhibitors, have reduced mortality rates among rheumatoid arthritis (RA) patients [2,3]. An analysis of data from a rheumatology registry reveals a substantial decrease in NSAID prescriptions for patients with inflammatory arthritis following the initiation of TNFi treatment. However, NSAID prescriptions for these patients remain higher than those in the general population [4]. These observations suggest a connection between joint discomfort in RA and synovial inflammation. However, recent studies indicate that pain levels in patients are not strongly linked to synovial inflammation [5,6]. Consequently, patients with inflammatory arthritis may require prolonged use of NSAIDs at elevated dosages. Therefore, when utilizing NSAIDs for analgesic purposes, the potential gastrointestinal complications they may induce must be duly considered.

RA patients have an increased risk of gastrointestinal perforations related to the disease [7,8]. The adverse gastrointestinal side effects linked to traditional NSAIDs (tNSAIDs) spurred the development of new NSAIDs that offer better gastrointestinal tolerability. This research ultimately led to the creation of selective COX-2 inhibitors, commonly called coxibs [9].

## 2 NSAIDs - effects and side effects

The term "non-steroidal anti-inflammatory drugs (NSAIDs)" refers to a broad range of chemical compounds that are among the most commonly used medications worldwide. These drugs treat pain, inflammation, and fever, addressing various conditions such as arthritis, headaches, migraines, menstrual pain, and common cold [9,10]. The therapeutic effect of NSAIDs stems from their ability to inhibit cyclooxygenase (COX), an enzyme crucial in the production of prostaglandins, which are involved in the development of inflammation and pain. COX exists in two isoforms: COX-1 and COX-2. COX-1 is consistently expressed in several types of cells, particularly platelets, while COX-2 is primarily inducible, meaning its expression increases rapidly in response to stress and inflammation. Since COX-1 inhibition can cause gastrointestinal ulcers and bleeding, and non-selective NSAIDs inhibit both COX enzymes, there's been a focus on developing selective COX-2 inhibitors [[9], [10], [11]]. In 1998 and 1999, the FDA approved celecoxib and rofecoxib. The Vioxx® Gastrointestinal Outcomes Research (VIGOR) trial, published in 2000, aimed to show that rofecoxib, a COX-2 specific inhibitor, reduced gastrointestinal adverse effects. The VIGOR study found that, in patients with RA, rofecoxib provided pain relief comparable to the tNSAID naproxen while significantly reducing upper and lower gastrointestinal events compared to naproxen [12]. However, the study also revealed an unexpected association between naproxen and a reduced risk of myocardial infarction in RA patients, leading to controversy and the eventual withdrawal of rofecoxib and other COX-2 inhibitors from the market [9,10]. The hypothesis was that COX-2 inhibitors might increase CV side effects by disrupting the balance between thromboxane A_2_ (TxA_2_) and prostacyclin (PGI_2_). TxA_2_, which promotes platelet aggregation and vasoconstriction, is regulated by COX-1, while PGI_2_, which inhibits platelet aggregation and promotes blood vessel dilation, is produced by COX-2. Although both tNSAIDs and selective COX-2 inhibitors suppress PGI_2_ production, coxibs also lead to an increased shift towards COX-1 activity [10,11]. After several years of controversy and the withdrawal of rofecoxib (Vioxx®) and parecoxib (Bextra®), the FDA issued a "Boxed Warning" in 2005, stating that all NSAIDs may increase the risk of heart attacks or strokes [9,13]. The European League Against Rheumatism (EULAR) has adopted these warnings and recommends that prescriptions of NSAIDs in RA and PsA should be with caution, especially for patients with documented CVD or in the presence of CVD risk factors [14]. In 2015, following a critical review of the data, the FDA released another warning highlighting several key points: First, there is an elevated risk of CV complications when using NSAIDs, particularly in individuals who are already at high CV risk. Second, the risk increases with prolonged use and higher doses. Third, there is also an increased risk of heart failure [9,13,15].

## 3 NSAIDs in patients with inflammatory arthritis

As shown in Table 1, several studies demonstrate that NSAIDs, known for their pain-relieving and anti-inflammatory properties, do not increase the risk of heart problems in patients with various types of inflammatory arthritis. This finding contrasts with the FDA's 2015 warning about potential CV risks associated with NSAID use.

**Table 1:** Selected references examining the cardioprotective effect of NSAID administration in patients with different inflammatory disorders.

Author, year	Disease	Study design	Treatment	N	Control group	N	Outcome	F/U in years	Result (p < 0,05) or (95 % CI)
Goodson NJ et al., 2009 [16]	new onset IP	primary care-based inception cohort	all NSAIDs	613	no NSAIDs	310	Risk of CVD mortality	10	baseline NSAID use: OR 0.54 (0.34–0.86)
Ferraz-Amaro I et al., 2009 [17]	RA	Prospective cohort	all NSAIDs	613	no NSAIDs	176	Risk of CV events	4	NSAID use: HR 0,78 (0,47 - 1,30)
Franklin J et al., 2010 [18]	recent onset IP	primary care based inception cohort	all NSAIDs	505	no NSAIDs	295	Risk of CVD	7	baseline NSAID use: RR 0,6 (0,3 - 0,9)
Bakland G et al., 2011 [19]	AS	regional cohort	all NSAIDs	677	no NSAIDs	2031	Risk of mortality	10	infrequent NSAID use: OR 4,35 (1,75 - 10,77)
Tsai WC et al., 2015 [20]	AS	Case-control study	all NSAIDs	232 cases	no NSAIDs	10165 controls	Risk of MACE	3	frequent NSAID use: OR 0,32 (0,06 - 1,57)
Haroon NN et al., 2015 [21]	AS	Population-based cohort	all NSAIDs	21473 AS	no NSAIDs	86606 without AS	Risk of vascular mortality	15	NSAID use: HR 0,1 (0,01 - 0,61)
Grigoriou A et al., 2016 [22]	RA	regional RA cohort	all NSAIDs	223	no NSAIDs	866	Risk of CV events	3,7	NSAID use: HR 0,77 (0,39 - 1,51)
Wu LC et al., 2016 [23]	AS	Population-based case-control study	all NSAIDs	346 cases	no NSAIDs	3766 controls	Risk of CAD	10	NSAID use: RR 0,96 (0,57 - 1,60)
			Celecoxib						Celecoxib use: OR 0,77 (0,50 - 1,17)
Tam HW et al., 2017 [24]	AS	Population-based cohort	Celecoxib	1208 AS	no NSAIDs	19328 without AS	Risk of CV events	10	Celecoxib use: HR 0,76 (0,66 - 0,86)
Hung YM et al., 2017 [25]	RA	Population-based cohort		6260		5168	Risk of CAD	10	
			Celecoxib	936	no NSAIDs				Celecoxib use: HR 0,63 (0,47 - 0,85)
			Etoricoxib	156	no NSAIDs				Etoricoxib use: HR 0,47 (0,26 - 0,84)
Lam SH et al., 2021 [26]	PsA	Retrospective cohort	all NSAIDs	139	no NSAIDs	61	Risk of MACE	8,8	NSAID use: HR 0,38 (0,15 - 0,96)
So H et al., 2023 [27]	RA	Population-based cohort		12233				8,7	
			tNSAIDs	8524	no NSAIDs	2858	Risk of MACE		tNSAID use: HR 0,83 (0,70 - 0,99)
			Cox-2 inhibitors	851	no NSAIDs	2858	Risk of MACE		Cox-2 inhibitors: HR 0,53 (0,36 - 0,77)
Fakih O et al., 2023 [28]	AS	Nationwide cohort		22929				8	
			all NSAIDs	19633	no NSAIDs	3296	Risk of MACE		NSAID use: SHR 0,39 (0,32 - 0,50)
			tNSAIDs	19297					tNSAID use: SHR 0,38 (0,30 - 0,47)
			Cox-2 inhibitors	3518					Cox-2 inhibitors use: SHR 0,56 (0,36 - 0,89)
Meng H et al., 2024 [29]	RA, PsA	Population-based cohort		13905				8,6	
			tNSAIDs	9747	no NSAIDs	3222	Risk of MACE		tNSAID use: HR 0,51 (0,44 - 0,58)
			COX-2 inhibitors	936					COX-2 inhibitors use: HR 0,60 (0,44 - 0,83)
	RA		tNSAIDs	8524		2858			tNSAID use: HR 0,85 (0,72 - 0,99)
			COX-2 inhibitors	851					COX-2 inhibitors use: HR 0,58 (0,40 - 0,83)
	PsA		tNSAIDs	1224		363			tNSAID use: HR 0,70 (0,40 - 1,24)
			COX-2 inhibitors	85					COX-2 inhibitors use: HR 1,10 (0,42 - 2,96)
Kao CM et al., 2025 [30]	RA	Population-based cohort	all NSAIDs	12166	no NSAIDs	166	Risk of MACE	5	NSAID use: HR 0,64 (0,48 - 0,87)

F/U, follow up; IP, inflammatory polyarthritis; RA, rheumatoid arthritis; AS, ankylosing spondylitis; PsA, psoriatic arthritis; CV, cardiovascular; CVD, cardiovascular disease; COX-2, Cyclooxygenase-2; OR, odds ratio; HR, hazard ratio; RR, relative risk; SHR, subhazard ratio; MACE, major adverse cardiovascular events; MACE was defined as the composite of myocardial infarction, ischaemic stroke, cardiovascular mortality, coronary artery bypass graft or procedures of coronary revascularisation; tNSAIDs, traditional NSAIDs.

First, studies indicate that individuals with inflammatory arthritis face a higher likelihood of developing heart disease [[31], [32], [33], [34]]. RA patients face a risk similar to that of patients with diabetes mellitus, comparable to the heart health risk typically seen in individuals being 10 years older [35]. The elevated risk of cardiovascular-related morbidity and mortality can be attributed to multiple factors, including inflammation and its interactions with disease activity and traditional CV risk factors [19,21,26,29].

Second, the studies referenced in Table 1 suggest a cardioprotective effect of NSAIDs, countering concerns about increased CV risk following prolonged use [20,28]. Third, two studies in Table 1 provide detailed information on the doses of NSAIDs used. In particular, they classify patients using celecoxib into three groups based on their defined daily dose (DDD): <1 DDD, 1–1.5 DDD, >1.5 DDD (1 DDD = 200 mg/day) [23,24]. Findings indicate that individuals using celecoxib at an average daily dose higher than 1.5 DDD (equivalent to greater than 300 mg) experience a reduced risk of coronary heart disease (CHD) compared to non-users of celecoxib, with an odds ratio of 0.34 (95 % CI: 0.13–0.89; P < 0.05). Notably, the highest dose was associated with the lowest risk of CHD [23,24].

The study conducted by Hung et al. [25] examined the impact of varying COX-2 inhibitors on the CHD risk among RA patients over a period of 10 years. Results showed that celecoxib and etoricoxib users had a lower incidence of CHD compared to those who did not use celecoxib.

Specifically, celecoxib's hazard ratio (HR) for CHD was 0.37 during the initial four years and increased to 1.29 thereafter. In contrast, etoricoxib users maintained a consistent HR of 0.47 (95 % CI: 0.26–0.84) throughout the study. The results of another study comparing the risk of ischemic stroke in RA patients [36] and the results of a meta-analysis on the use of NSAIDs in ankylosing spondylitis [37] suggest that both COX-2 inhibitors, celecoxib and etoricoxib, can reduce pain, inflammation, and the risk of a CV event, whereby the better efficacy of etoricoxib also correlates with comparative data on effective treatment of postoperative acute pain [38].

The studies presented in Table 1 explore the CV risks faced by patients with inflammatory arthritis who are prescribed NSAIDs. Long-term use of NSAIDs has been associated with a lower CV risk in these patients, which is in stark contrast to the experience of NSAID usage in the general population, where prolonged use is linked to an increased risk [39]. However, NSAIDs are typically viewed as symptomatic, adjunctive medications for patients with RA, while they are considered first-line therapy in ankylosing spondylitis (AS). Due to their association with reducing spinal ossification and delaying disease progression, it is not surprising that most studies in Table 1 focus on ankylosing spondylitis [15,37,39].

## 4 NSAIDs for OA and RA with different CV risks?

In a meta-analysis, Schieir et al. [40] examined the risk of myocardial infarction in patients with different types of arthritis. The relative risk for RA patients was found to be higher (RR: 1.69, 95 % CI 1.50 to 1.90) compared to patients with osteoarthritis (OA) (RR: 1.31, 95 % CI 1.01 to 1.71). However, the risk for ankylosing spondylitis (AS) patients (RR: 1.24, 95 % CI 0.93 to 1.65) did not differ significantly from that of OA. Interestingly, when analysing the CV risk associated with NSAIDs in these patient populations, it was observed that RA patients with NSAIDs exhibit substantially lower CV risk than control subjects (HR 1.22, 95 % CI 1.09 to 1.37 compared to 1.51, 95 % CI 1.36 to 1.66, p < 0.01) [41]. This finding was explored in more detail in a separate publication [42].

Nonetheless, patients with RA showed a higher rate of CV events compared to control groups, mainly composed of OA patients [41]. A review of existing research on this topic indicates that only two studies, the MEDAL [43] and PRECISION [44] trials, specifically investigated the CV risk of NSAIDs in patients with OA and RA as a primary objective. The MEDAL program compared the CV safety of the COX-2-specific inhibitor etoricoxib with diclofenac, while the PRECISION study compared celecoxib with ibuprofen and naproxen. Both studies aimed to demonstrate that these COX-2 inhibitors have comparable CV safety to tNSAIDs, with fewer gastrointestinal and kidney side effects. Notably, RA patients consistently experienced a higher incidence of thrombotic CV events compared to those with OA [43,45]. However, the findings revealed no statistically significant differences in the study's CV endpoints between the studied coxibs and tNSAIDs [43,44].

A recent large-scale meta-analysis concluded that the short-term use of all NSAIDs is associated with an increased risk of myocardial infarction, with odds ratios ranging from 1.24 for celecoxib to 1.58 for rofecoxib in the general population [46]. Most of the population studied were OA patients, with RA patients being underrepresented, comprising only 1.8–5.5 % of the sample. Roubille's meta-analysis is one of the few that explicitly addresses CV risk in RA patients, showing a modest increase in CV events associated with NSAID use (RR 1.18; 95 % CI 1.01–1.38), particularly for rofecoxib, which is no longer on the market. However, this increase was not observed with celecoxib or other tNSAIDs [47].

While RA patients have an increased CV risk compared to OA patients, they exhibit a lower CV risk when treated with NSAIDs compared to OA patients. The reasons for these differing observations become clearer when considering the various factors influencing the CV systems of the two types of arthritis. It is essential to recognize that the risk of developing CV disease is increased by multiple factors in patients experiencing pain and requiring NSAIDs, regardless of whether they have RA or OA [42]. In a review, Corrao et al. [48] described several cardiometabolic and other related conditions that contribute to increased CV morbidity and mortality in RA patients. The CV health of individuals with RA and OA is primarily influenced by four components: established CV risk factors (including age, hypertension, dyslipidaemia, obesity, smoking, and diabetes), metabolic syndrome, systemic inflammation, and the patient's pain status [42,[49], [50], [51]]. Traditional CV risk factors help estimate the risk of CV disease in both RA and OA; however, they are less effective in distinguishing differences in CV morbidity and mortality between the two conditions [42,45].

In a multicentre study examining the prevalence of metabolic syndrome in RA and OA patients, it was found that individuals with OA had a 1.6-fold higher prevalence of metabolic syndrome than those with RA. Patients diagnosed with metabolic syndrome exhibited an increased waist circumference, and elevated levels for systolic blood pressure, fasting blood glucose, and triglyceride levels, in addition to increased inflammatory marker levels [(C-reactive protein (CRP), and erythrocyte sedimentation rate (ESR)] compared to those who did not meet the criteria for metabolic syndrome [49].

A different perspective emerges when examining the role of systemic inflammation in patients with OA and RA. In discussions about the differing effects of NSAIDs on these patients, authors frequently refer to "non-inflammatory arthritis" and "inflammatory arthritis" [42]. However, both types of arthritis are associated with inflammation. Different elevations of specific markers in the serum and synovial fluid of patients allow for differentiation between the two types.

Elevated levels of interleukin 19 (IL-19) have been found in patients with both RA and OA, suggesting that IL-19 may play a role in developing joint inflammation. Additionally, RA patients tend to have higher levels of serum CRP, tumour necrosis factor-alpha (TNF-α), interleukin-6 (IL-6), IL-20, and IL-24 compared to those with OA, indicating a greater state of systemic inflammation [48,50,51]. Systemic inflammation is significant in determining the risk of CV morbidity and mortality in RA, a topic that has been thoroughly explored in various studies [15,42,47,48].

For instance, data from the JUPITER study indicate that inflammation and elevated low-density lipoprotein cholesterol (LDL-C) levels contribute to atherosclerosis and an increased risk of CV disease [52]. Interestingly, a "paradoxical interaction" has been observed in RA patients, where lower levels of LDL are linked to a higher CV risk. In systemic inflammation, reduced cholesterol levels are common; however, during anti-inflammatory treatment for RA, LDL levels may increase without a corresponding rise in CV event risk [48]. This paradox may also explain the findings of Kao et al. [30] that suggest hyperlipidaemia could be a protective factor for elderly RA patients.

Assessing the connection between inflammation and heart disease risk in RA patients using existing risk scores presents challenges. While these scores effectively predict CV risk in the general population, they often underestimate risk in individuals with inflammatory arthritis. To address this, the European Rheumatism League recommends multiplying standard CV disease risk estimates for RA patients by 1.5, which only partially enhances prediction accuracy [48,53].

The ongoing nature of pain, which is more prevalent in OA patients than in RA patients [45], is another factor contributing to increased CV risk. Pain associated with OA drives the need for NSAIDs, leading to the hypothesis that these medications may elevate CV risk [42,54]. Cohort studies have shown a correlation between chronic pain, depression, and CV disease, highlighting shared genetic components and underscoring chronic pain as a CV risk factor [55]. A detailed examination of pain in OA and RA patients reveals further differences: OA patients report a significantly higher incidence of neuropathic pain (60.9 %) compared to RA patients (36.7 %), and OA patients also experience more severe pain [56]. In this context, it is noteworthy that a significant proportion of RA patients also experience OA [57]. These findings emphasize the importance of understanding pain in different forms of arthritis and suggest that therapeutic responses to NSAIDs may vary [6].

In some cases, the administration of NSAIDs as the sole treatment modality for arthritis patients with complex pain problems can result in suboptimal pain management and reduced functionality. This, in turn, can have a detrimental effect on the patients’ quality of life [6]. However, most RA patients benefit from NSAIDs, as these drugs reduce inflammation, joint pain, and swelling. As a result, they help lower CV risk and depression rates while improving quality of life [55,56]. Therefore, it is not surprising that prolonged NSAID use in ankylosing spondylitis (AS) patients has been associated with cardioprotective effects. Indeed, NSAID administration is a key aspect of first-line therapy for symptomatic AS patients [[19], [20], [21],^23^,^24^,^28^].

Given the study results, it is essential to reevaluate the findings of the PRECISION study. Announced as the first research focusing on OA and RA patients with high CV risk - who often require long-term NSAID treatment - the study examined the CV safety of these therapies [58]. This 10-year study, with an average treatment duration of 20 months, demonstrated that moderate doses of celecoxib were not inferior to tNSAIDs, such as ibuprofen or naproxen, concerning CV risk [44]. The results from a time-to-event analysis within the PRECISION study indicated that adverse CV events occurred less frequently in the On-Treatment Population compared to the Intention-to-Treat Population. However, the On-Treatment analyses had a more extended follow-up period of 43 months compared to 30 months for the Intention-to-Treat Population. Since data from the On-Treatment Population represent a more conservative approach [43], and considering that the PRECISION study included patients with arthritis and high CV risk [58], it can be inferred that NSAID administration will likely reduce CV risk in these patients. The low rate of CV events observed in the PRECISION study - approximately 1 % per year - supports this inference, indicating a reduced CV risk with NSAID treatment. This finding is further corroborated by studies presented in Table 1 and aligns with the conclusions of other authors [15,22,42,59].

## 5 Does the study design influence the CV risk of NSAIDs?

RA is a known risk factor for heart disease, so rheumatologists focus on managing chronic inflammation and disease activity along with traditional CV risk factors [60]. Regarding CV safety, the medications prescribed are also crucial for rheumatologists. Research indicates that the CV risk in RA patients increases in a dose-dependent manner with long-term use of NSAIDs.

**Fig. 1:**
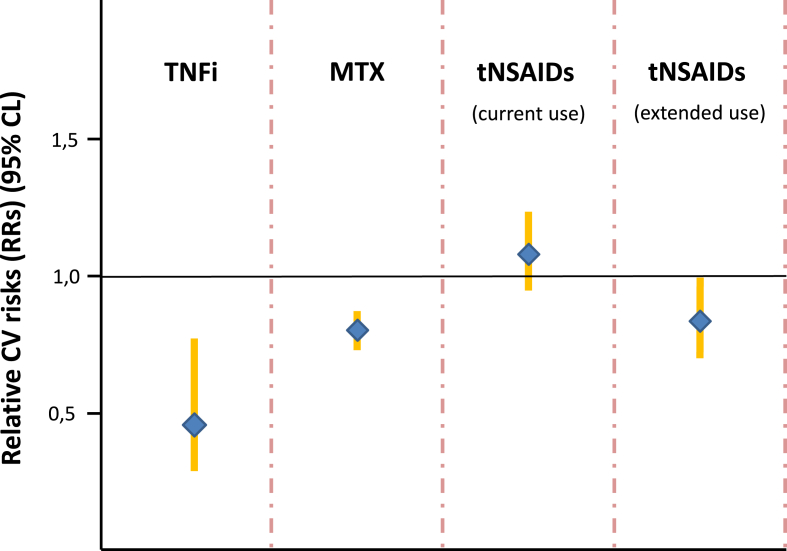
The figure provides a visual representation of the relative risks (RRs) for CV events in patients with RA who are being treated with TNF inhibitors (TNFi)[63], methotrexate (MTX) [64], and tNSAIDs [27, 47]. These RRs are derived from meta-analyses of observational studies or cohort analyses.

However, observations regarding NSAIDs also reveal that shifts in study design can alter results. In a meta-analysis by Roubille et al., NSAIDs were associated with an elevated risk of CVEs [RR 1.18 (1.01–1.38)] in RA patients (Fig. 1), with this increased risk primarily linked to rofecoxib [47]. However, a population-based retrospective cohort study covering 8.7 years found a reduced risk of MACE [HR 0,83 (0,70–0,99)] with tNSAIDs (Fig. 1) [27]. Another nested case-control study investigating myocardial infarction risk in patients with spondyloarthritis (SpA) and OA who took tNSAIDs indicated that SpA patients currently using diclofenac had a two-to threefold higher risk of MI, while no increased risk was found for naproxen [61]. Research by Kao and colleagues contributed significant findings: a case-control study involving patients newly diagnosed with ankylosing spondylitis (AS) showed that NSAIDs do not reduce the risk of MACE [62].

In contrast, a cohort study identified NSAIDs as potentially cardioprotective in RA patients [30]. Similarly, the results of cohort studies listed in Table 1 suggest no significant increased risk associated with all tNSAIDs and COX-2-specific inhibitors in patients with AS, indicating a possible cardioprotective effect of these medications. Only one AS study (Table 1, a case-control study) reported a specific finding: a reduced risk of CHD with coxibs (celecoxib and etoricoxib) but not with naproxen or diclofenac, emphasizing the importance of study design [23]. Of note, observational studies are more susceptible to bias and confounding than randomized controlled trials [47].

Even with methotrexate and TNF inhibitors differing results concerning CV events in RA patients were reported [60]. Various meta-analyses consistently demonstrate a lower risk of all CV diseases in RA patients treated with TNF inhibitors [63] and methotrexate [64]. Specifically, TNF inhibitors reduce this risk by 54 %, greater than the 21 % reduction associated with methotrexate (Fig. 1). Another meta-analysis showed that TNF inhibitors or methotrexate decreased CV events by 30 % and 28 %, respectively, indicating comparable risk reductions [47]. However, a network meta-analysis examining the risk of major adverse cardiovascular events (MACE) in immune-mediated inflammatory diseases reached a different conclusion. Based on randomized controlled trials, this analysis found that TNF inhibitors increase the risk of MACE compared to placebo, with an odds ratio of 2.49 (1.14–5.62) [65].

Taken together, study design is crucial since it may influence various outcomes. Furthermore, inadequate follow-up may have masked any effects of NSAID treatment on CV event rates in patients with inflammatory arthritis.

## 6 Is it time to rethink the CV risk of NSAIDs in inflammatory arthritis?

Although the use of NSAIDs is associated with a higher risk of CV disease in the general population [46], data presented in Table 1 and a meta-analysis exploring the relationship between COX-2 inhibitor use and cardiovascular events in patients with ankylosing spondylitis (AS) suggest that there is no increased risk for patients with inflammatory arthritis [RR 0.48 (0.33–0.70)] [39].

It's time to reconsider the use of NSAIDs in patients with inflammatory arthritis for a few compelling reasons. First, the prevailing notion that COX-2-specific NSAIDs contribute to CV risk by disrupting the balance between prostacyclin (COX-2) and thromboxane A_2_ (COX-1), thereby creating a "prothrombotic state," is often cited. However, a controversial safety update highlights that this theory originated from a study indicating that COX-2-specific NSAIDs increased thromboxane metabolites in urine while decreasing prostacyclin metabolites. This theory conveniently overlooks that paracetamol produced similar results in a previous study with the same methodology, yet it was not labelled as prothrombotic [66].

Secondly, experiments have yet to confirm the theory of “imbalance of prostaglandin homeostasis.” Findings suggest that under physiological conditions, COX-1 - rather than COX-2 - drives the production of prostacyclin in the CV system. Furthermore, urinary prostacyclin measurements do not accurately reflect endogenous prostacyclin production within systemic circulation [67].

Thirdly, research on the role of prostacyclin as both a physiological and pathological mediator, as well as a therapeutic agent, has revealed its significance as an inflammatory mediator and its homeostatic functions in the CV system [68]. Prostacyclin (PGI_2_) has paradoxical effects in inflammatory diseases: it offers protection against inflammation in atherosclerosis but may promote inflammatory responses in rheumatic diseases such as RA and OA [69]. This conflicting nature of prostacyclin can also account for the varying results observed in different studies. For instance, Salpeter's meta-analysis found no association between NSAID use and CV risk in patients with joint disease [OR 0.6 (0.2–1.7)], while Alzheimer's patients without pain or inflammation were at a higher risk [OR 1.6 (0.9–2.7)] [66]. The studies listed in Table 1 support the findings of the Salpeter meta-analysis [70] and underscore the "paradoxical role of prostacyclin," prompting a focus on the primary purpose of NSAIDs: alleviating pain and inflammation [9].

The authors of the first cohort study, which initially suggested a "paradoxical relationship between NSAID use and reduced CV disease mortality" carefully scrutinized their results prior to publication [16]. The majority of the studies included in Table 1 examined not only the association between NSAID use and CV disease risk but also a comprehensive range of additional risk factors. The following aspects were examined in detail: In earlier studies, patients who did not receive NSAIDs often received glucocorticoids as an alternative therapy. Since glucocorticoids can increase atherogenesis and potentially bias CV results in favour of NSAIDs, this effect was therefore excluded [16]. The data in the literature suggest that there are gender-based differences among RA patients. Female patients have higher disease activity and a greater need for biologics, which are less effective in this group, while male patients have a higher CV risk [21,25,[27], [28], [29],^72^]. However, studies report no gender-based differences in NSAID use (Table 1). Multivariable models that adjusted for different CV risk scores identified inflammatory burden (as measured by the Bath Ankylosing Spondylitis Disease Activity Index [BASDAI] and inflammatory markers) as a significant risk factor for CV events. In contrast, the use of NSAIDs, statins, and DMARDs was shown to reduce CV risk in patients with axial SpA [21,71]. For RA patients, NSAIDs are particularly beneficial for older individuals and those with high CV risk [30]. Studies involving patients with AS or RA have demonstrated that COX-2 inhibitors are linked to a lower risk of coronary heart disease (CHD), with etoricoxib proving superior to celecoxib, which showed a lower CV risk only at higher dosages [[23], [24], [25]].

In contrast, the current ACR [73] and EULAR [14] recommendations regarding the use of NSAIDs in RA and OA patients with CV disease impose several restrictions. These guidelines are primarily based on FDA warnings [13] and on studies with methodological limitations [46,75]. A Cochrane Review did not find any study addressing NSAID use in RA patients with CV comorbidity. They identified one trial that reported evidence in mixed populations (including both RA and OA) taking either diclofenac or etoricoxib. In this study, the presence of CV disease increased the likelihood of a further CV event three-fold [74]. However, these studies do not reflect the current state of scientific knowledge or modern RA treatment strategies, including a Treat-to-Target approach [44,45]. Nevertheless, secondary publications consistently recommend NSAIDs at the lowest effective dose and for the shortest possible duration. They also advise avoiding NSAIDs in patients with significant CV comorbidities [9,10].

## 7 Conclusion

It's time to reconsider the use of NSAIDs in patients with inflammatory arthritis for a few compelling reasons. Even though a few studies suggest an increased risk for CV events due to NSAID use in these patients, there exist various studies underlining a protective effect of tNSAIDs as well as coxibs on CV events. It has to be considered that the study design is crucial for interpretation of the results since it may influence various outcomes. The somewhat “protective” effect of NSAIDs has not been demonstrated in other pain syndromes where NSAID therapy is indicated. However, in patients with inflammatory arthritis and OA the anti-inflammatory effect of NSAIDs is most probably the relevant factor in reducing CV events in these patients. This study aims to critically synthesize and interpret the existing literature to highlight discrepancies between current guideline recommendations and recent findings, particularly in the context of inflammatory arthritis. These findings should be supplemented with recent, high-quality studies and incorporated into a rigorous meta-analysis to inform future treatment recommendations for NSAID use in patients with RA. Nonetheless, it remains to be seen to what extent the regulatory authorities will take the findings of these studies into account and modify the box warning.

## Contributions

Both authors contributed equally to the study. GZ made the tables and figures and wrote the first draft. CB had access to all the data and was responsible for accuracy and for approving final publication. Both authors read and approved the final version.

## Declaration of competing interest

The authors declare the following financial interests/personal relationships which may be considered as potential competing interests:Gerhard Zingler (retired employee) reports a relationship with MSD Germany that includes: employment. If there are other authors, they declare that they have no known competing financial interests or personal relationships that could have appeared to influence the work reported in this paper.
